# Elemental Spatiotemporal Variations of Total Suspended Particles in Jeddah City

**DOI:** 10.1155/2014/325492

**Published:** 2014-02-18

**Authors:** Mohammad W. Kadi

**Affiliations:** ^1^Department of Chemistry, Faculty of Science, King Abdulaziz University, P.O. Box 80203, Jeddah 21589, Saudi Arabia; ^2^Centre of Excellency in Environmental Studies, King Abdulaziz University, P.O. Box 80216, Jeddah 21589, Saudi Arabia

## Abstract

Elements associated with total suspended particulate matter (TSP) in Jeddah city were determined. Using high-volume samplers, TSP samples were simultaneously collected over a one-year period from seven sampling sites. Samples were analyzed for Al, Ba, Ca, Cu, Mg, Fe, Mn, Zn, Ti, V, Cr, Co, Ni, As, and Sr. Results revealed great dependence of element contents on spatial and temporal variations. Two sites characterized by busy roads, workshops, heavy population, and heavy trucking have high levels of all measured elements. Concentrations of most elements at the two sites exhibit strong spatial gradients and concentrations of elements at these sites are higher than other locations. The highest concentrations of elements were observed during June–August because of dust storms, significant increase in energy consumption, and active surface winds. Enrichment factors of elements at the high-level sites have values in the range >10~60 while for Cu and Zn the enrichment factors are much higher (~0–>700) indicating that greater percentage of TSP composition for these three elements in air comes from anthropogenic activities.

## 1. Introduction

Atmospheric particulate matter's (PM) role in human health is increasingly being recognized. Specific health effects now are known to have a direct link to exposure to PM. For example, pulmonary and cardiac diseases connection to PM is well established [1, 2]. PM has a direct effect on the radiative balance of the earth's atmosphere by scattering or absorbing radiation and acts as cloud condensation nuclei [3–7]. Many atmospheric chemical reactions are often mediated via PM surface properties. Chemical composition is an important property of PM as chemical constituents affect the way particles behave. Effects that PM induces on biota and the environment is generally governed by its chemical composition. For example, the presence of elevated levels of Pb in PM would increase health risks associated with this element that include enzymes inhibition and strong toxicity to the brain [8, 9].

Particle size is another important property of PM as it is well established now that those smaller size particles, namely, PM10, PM2.5, and smaller ones, deposit more easily deep into lung tissues causing the adverse health effects. Many researchers also measure total suspended particles (TSP) as an important measure of PM. In fact in many instances data from TSP is very important to establish indicators for compliance to standards set by regulating authorities [10].

Numerous studies on PM across many cities and countries around the world have been conducted. It is important to study PM of the local environment as factors affecting its concentrations and behavior make extrapolation to other locations impractical. Nguyen et al. reported their findings on concentrations of TSP-bound metals in Seoul, Korea. In this study, two major sources of metals in PM were identified, resuspension from soil and vehicular emissions [11]. Malandrino et al. observed temporal trends of 15 elements over a long period in Turin, Italy [12]. In India, Basha et al. assessed heavy metal content of TSP of a coastal industrial town [13]. Substantial contribution from anthropogenic sources was identified as a reason for increased elemental content of TSP. Schleicher et al. investigated the temporal variability of trace elements of urban PM from Beijing [14]. They concluded that industrial and traffic related sources played a major role in elemental composition of PM. Mmari et al. reported elemental and water soluble inorganic ions concentrations on TSP of the atmosphere near Dar es Salaam, Tanzania [15]. It is evident from these and other studies that there is so much to be learned about composition, behavior, and effects of PM on humans and the environment.

This work reports spatial and temporal distribution of 15 elements (Al, Ba, Ca, Cu, Mg, Fe, Mn, Zn, Ti, V, Cr, Co, Ni, As, and Sr). This paper represents one part of a series designed to achieve a better understanding of the fundamental characteristics of PM in Jeddah city in an effort to design best ways to reduce emissions and mitigate potential harmful pollutants and to establish complete up to date data.

## 2. Experimental

### 2.1. Sampling

**Figure 1:**
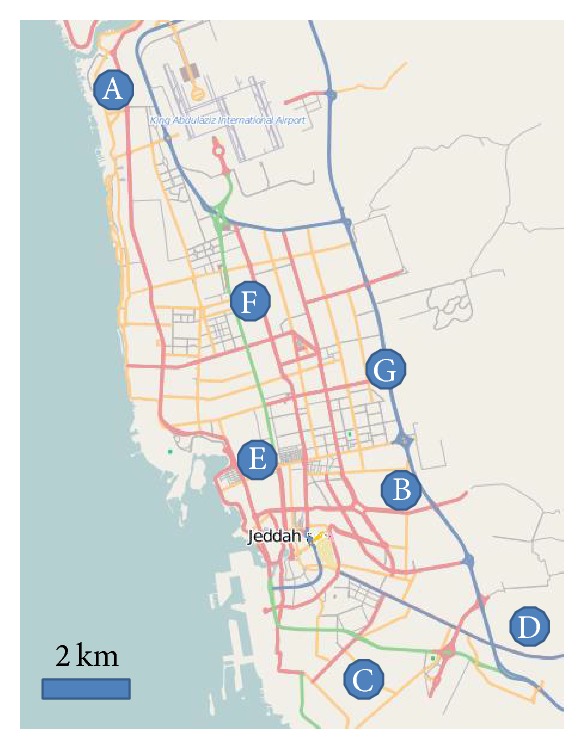
Sampling sites and their relative locations on the map of Jeddah city.

TSP samples were collected from seven sampling sites spanning from north to south of the city. Crowded residential-commercial activities, residential areas, heavy traffic, industrial activities, and areas with heavy trucking activities are represented in these sample sites.  Table 1 summarizes sampling site information and characteristics. Sampling locations and relative positions are also indicated on the map of the city in Figure 1. TSP samples were collected on binderless Whatman 41 filter papers using high-volume Thermo Scientific Samplers Model MFC-TSP. This sampler utilizes a mass flow controlled system for TSP sampling and is designed to meet US EPA reference method for determination of total suspended particulate concentrations. A collection period of 24 hours is more than enough for good analytical results as was demonstrated in trials prior to actual sampling. Samplers were calibrated according to manufacturer's recommendation and in compliance with EPA procedures.

### 2.2. Apparatus and Material

After sampling, aerosol samples were in sealed polyethylene bags and transferred to the lab for analysis. Analytical reagent grade chemicals were used in all preparations. Solutions were prepared in deionized water produced in the lab utilizing a Milli-Q plus system (Millipore, Bedford, MA, USA). This system produces deionized water with a resistivity value of less than 18.2 MΩ·cm at 25°C. Aerosol samples were acid-digested and elemental content was determined employing ICP-OES (Optima 4100 DV) and ICP-MS (Sciex Elan DRCII, USA) Perkin-Elmer spectrometers. The ICP instruments were optimized daily before measurements and operated as recommended by the manufacturer.

### 2.3. Total Acid Digestion and Analysis of the Aerosol Samples

Total acid digestion procedure used for elemental analysis of aerosol samples was performed as follows. Transfer 1/4 of the filter paper loaded with PM into a 30 mL PTFE vial. Add 7.5 mL of conc. HNO_3_, close the bottle, and leave the mixture for 24 hours. The second step involves heating on a hot plate until evolution of NO_2_ fumes ceases. After cooling, add ~1.5 mL HF and leave the sample for additional 24 hours. Remove the lid and heat the sample until near dryness. Add 1 mL conc. HNO_3_ and heat the sample to near dryness; this step is repeated twice. Add 7 mL 2% HNO_3_ in several portions and transfer the sample analytically to a centrifuge test tube. Transfer the solution to a 25 mL polyethylene volumetric flask and store for analysis [16]. IAEA-soil-7 certified reference material (CRM) and multielement standard solution were used to certify the validity of the analytical procedures. The CRM sample was digested in a fashion similar to that of aerosol samples.

## 3. Results and Discussion

Jeddah is the second largest city in Saudi Arabia after the capital Riyadh. It is located west of the country on the shore of the Red Sea with urban areas concentrated on the shoreline that extends to about 60 km. Jeddah is an ancient city that became a major commercial hub and a gateway to Makkah, one of the most travelled-to cities throughout history. With a population approaching 4 million, the biggest seaport on the Red Sea, one of the busiest airports in the region, and ever expanding industrial activities, pollution studies are important in order to develop strategies for pollution control and abatement measures.

In this study, 15 elemental concentrations were followed for a period of one year at seven sites in the city; minimum and maximum observed concentrations along with mean and standard deviations are represented in Table 2. High standard deviation values indicate large variations in concentrations between sites, as it is clear for Al, Ca, and Fe.

### 3.1. Spatial and Temporal Trends of Metals in TSP

**Figure 2:**
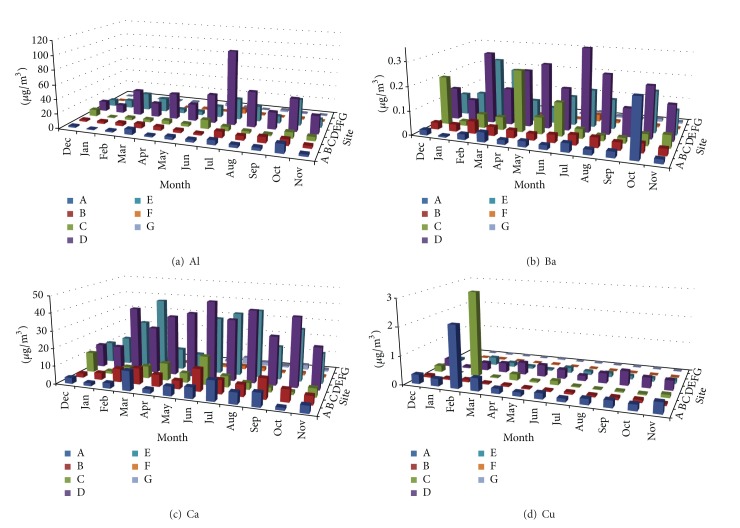
Spatial and temporal distribution of Al, Ba, Ca, and Cu.

**Figure 3:**
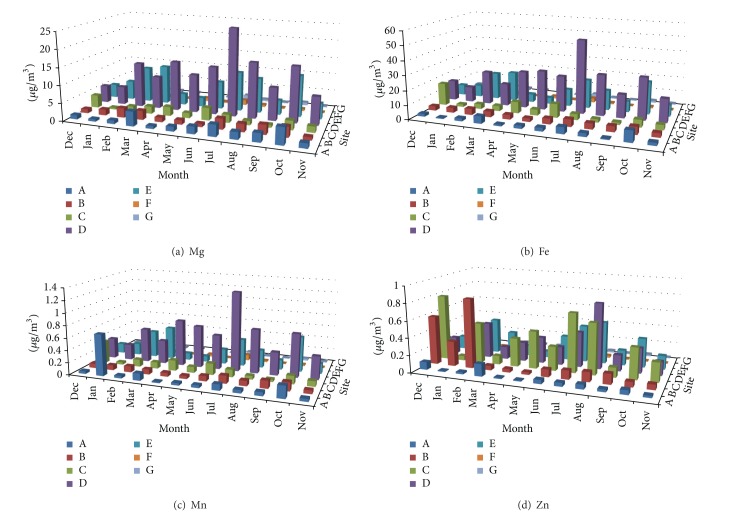
Spatial and temporal distribution of Mg, Fe, Mn, and Zn.

**Figure 4:**
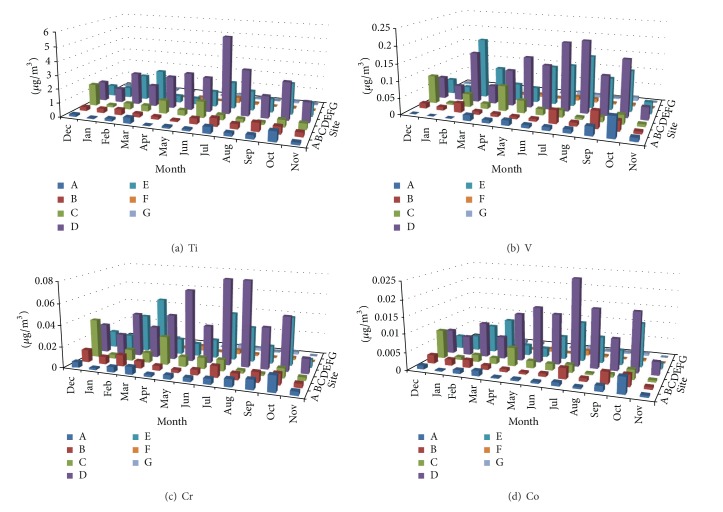
Spatial and temporal distribution of Ti, V, Cr, and Co.

**Figure 5:**
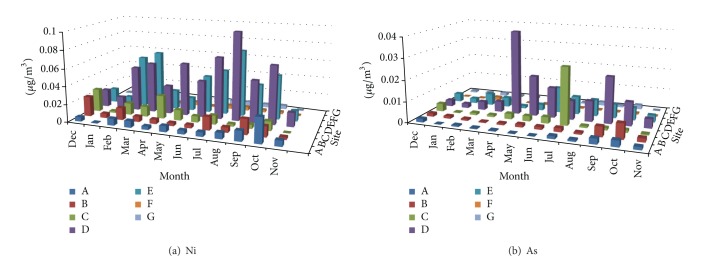
Spatial and temporal distribution of Ni and As.

**Figure 6:**
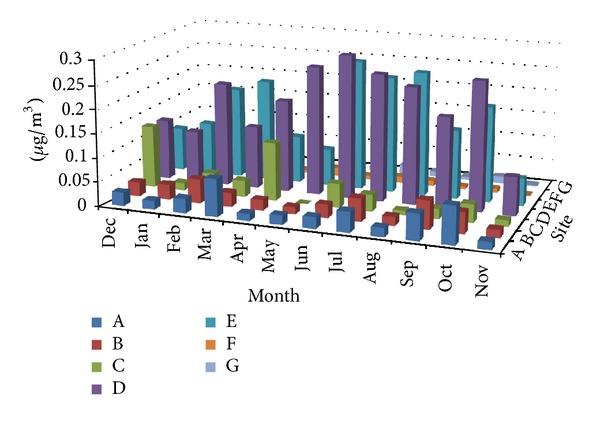
Spatial and temporal distribution of Sr.

Measured concentrations of Al, Ba, Ca, Cu, Mg, Fe, Mn, Zn, Ti, V, Cr, Co, Ni, As, and Sr in TSP samples at the seven sampling sites during the sampling campaign are shown in Figures 2, 3, 4, 5, and 6. In the 3D representation, a column represents the observed concentration of an element at locations A, B, C, D, E, F, and G at various times of measurement.

#### 3.1.1. Spatial Behavior

One striking feature of Figures 2–6 is that sites D and E stand out as the two sites with high concentrations for all measured elements almost each time with few exceptions. Site D is located near one of the oldest roads in the country. Major percentage of heavy-duty transportation passes through this road. Ceramics and tile industries are also concentrated around this road along with many warehouses and various workshops. Site E is near the city center, which leans to the south, with huge number of commercial shops and narrow roads. The commercial activity in this area is far apart from the spacious modern malls north of the city. Shops here are small and large in numbers. The observation of high concentrations at locations D and E shows the effect of localized activities on levels on air pollution. That is, pollutants might not spread out as quickly but levels of concentrations will remain higher than other parts of the city due to localized sources and resuspension. Constant feeding of pollutants into the local environment will increase metal concentrations in the soil within and surrounding the site. The soil will act as a reservoir of pollutants, which are then resuspended as a direct result of vehicular activities and surface winds.

At sites A, B, C, D, and E, Ca showed relativly high concentrations. This is probably because of increased construction activities at sites A, B, and C adding to that cement-related industries around sites D and E. Site C (the industrial city) shows comparable concentrations of Ba and Zn to those of sites D and E; this could be because of heavy and light transportation into and out of the industrial area.

#### 3.1.2. Temporal Behavior

Inspection of Figures 2–6 reveals that the highest concentrations of elements in general occur during the period June–August. October also shows high concentrations in many instances. Many authors reported increased PM elemental concentrations in winter with lowest reported levels in summer. Reasons for the winter increase include increased energy consumption, thermal inversion that occurs in winter [12], and stronger winds [17]. In some regions, summer concentrations are higher. Singla et al. reported higher summer of concentrations of TSP because of dust storms [18]. High temperatures in summer result in a huge increase in energy consumption used in air-conditioning. On the other hand heating is not required in fall and winter because of mild temperatures. The high consumption of fossil fuels explains higher PM elemental concentrations during summer season. The second reason for the concentration increase is the active surface winds and dust storms in the summer.

### 3.2. Enrichment Factors

**Figure 7:**
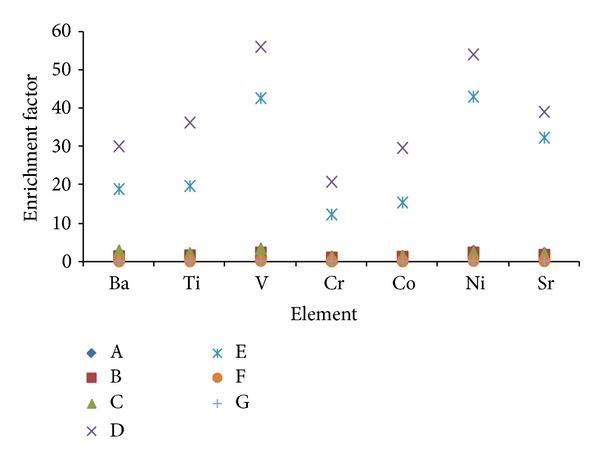
Enrichment factors of Ba, Ti, V, Cr, Co, Ni, and Sr at the 7 sampling sites.

**Figure 8:**
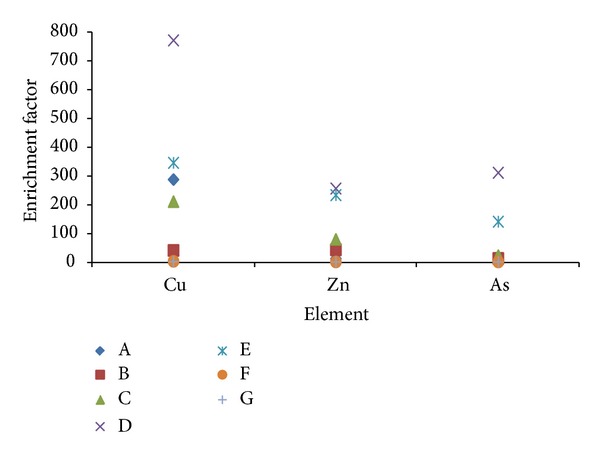
Enrichment factors of Cu, Zn, and As at the seven sampling sites.

Analysis of elemental concentration ratios in TSP are frequently used to allocate sources of trace elements [19]. One approach for this is to compare the ratios in samples with those in a likely source material by calculating enrichment factors (EF). Enrichment factors relative to upper continental crust composition are used to understand the relative dominance of crustal or anthropogenic sources for an element in aerosols [19, 20]. The enrichment factor of an element in PM can be calculated using the equation

(1) $$\begin{array}{c} \text{EF}={\left[T\right]}_{\text{air}}/{\left[R\right]}_{\text{air}}/{\left[T\right]}_{\text{crust}}/{\left[R\right]}_{\text{crust}} \end{array}$$

where [*T*]_air_ and [*R*]_air_ are the concentrations of the element and the reference element in the atmosphere, while [*T*]_crust_ and [*R*]_crust_ are the mean concentrations of the elemental component and the reference element in the earth's crust [19–21]. By convention, if EF approaches unity, the earth's crust is the predominant source. If EF > 1, this suggests that a significant fraction of the element is controlled by input from noncrustal sources. The element in this case is referred to as enriched element [21]. Enrichment factors are useful in rationalizing data; however, they should be interpreted cautiously as regional variations in the mineralogy of the earth's crust are generally not considered. In the present study, Al, the most abundant element in the earth's crust, was used as a reference element. Calculated enrichment factors of elements in this study are represented in Figures 7 and 8. Enrichment factors of Ba, Ti, V, Cr, Co, Ni, and Sr lie within the range ~0–60 as shown in Figure 7. Sites A, B, C, F, and G have very small enrichment-factor values indicating predominant crustal content of all these elements. However, enrichment-factor values of Ba, Ti, V, Cr, Co, Ni, and Sr at sites D and E have higher values in the range >10~60 which is a clear indicator of anthropogenic sources. Enrichment factors for Cu, Zn, and As show similar behavior except that values of the factors of these elements are much higher (~0–>700). This indicates that greater percentage of these three elements concentration in the air comes from anthropogenic activities. Cu is the only element that showed high enrichment factors at locations other than D and E. This is because observed Cu concentration in the month of February at sites A and C was high. An isolated event in which Cu was released at these two sites might have contributed to this observation. Zn shows relativly high values of enrichment at locations B, C, D, and E, all of which are characterized as sites with heavy traffic. Mechanical abrasion of tires is known to be a major source of Zn along with other sources [22].

## 4. Conclusion

Elemental composition of total suspended particulate matter in Jeddah city was studied. Al, Ba, Ca, Cu, Mg, Fe, Mn, Zn, Ti, V, Cr, Co, Ni, As, and Sr concentrations were followed at seven sampling sites. Spatial and temporal trends of levels of these elements in TSP were investigated. Two sampling sites with increased anthropogenic activities stand out as having high elemental concentrations. Local activities greatly affect levels of pollution within certain parts of the city. Concentrated efforts in cleanup and environmental control in areas with high elemental concentrations will reflect on air quality of the entire city.

## Figures and Tables

**Table 1:** Sampling site characteristics.

Site	Site characteristics
A	(i) North of the city
	(ii) On the red sea shore
	(iii) Residential and recreational activities

B	(i) Middle of the city to the south
	(ii) King Abdulaziz University main campus
	(iii) Near busy roads

C	(i) The industrial area, south of the city
	(ii) Various light-industrial activities and factories

D	(i) South-east of the city
	(ii) Busy roads with many workshops
	(iii) Very busy heavy trucking
	(iv) Ceramic tiles, construction materials

E	(i) City center
	(ii) Crowded with heavy population and slow traffic
	(iii) Major commercial center

F	(i) Northern part of the city
	(ii) Crossroads and residential area

G	(i) At the edge of a major highway in Jeddah
	(ii) Residential area

**Table 2:** Minimum and maximum measured concentrations in TSP samples (*μ*g/m^3^).

Element	Min.	Max.	Mean	Std. dev.
Al	0.30	102.9	10.02	15.1
Ba	0.004	0.46	0.077	0.09
Ca	0.51	44.4	11.2	12.5
Cu	0.007	3.5	2.0	0.45
Mg	0.16	24.9	3.8	4.7
Fe	0.37	51.3	7.2	9.2
Mn	0.007	1.3	0.18	0.24
Zn	0.006	0.82	0.17	0.24
Ti	0.031	5.56	0.74	0.95
V	0*	0.213	0.042	0.053
Cr	0.0004	0.081	0.014	0.018
Co	0*	0.027	0.004	0.005
Ni	0*	0.099	0.017	0.021
As	0*	0.041	0.0037	0.006
Sr	0*	0.29	0.07	0.083

*Under the detection limit.
